# The Stability and Complexity Analysis of a Low-Carbon Supply Chain Considering Fairness Concern Behavior and Sales Service

**DOI:** 10.3390/ijerph16152711

**Published:** 2019-07-30

**Authors:** Qiuxiang Li, Xingli Chen, Yimin Huang

**Affiliations:** 1 Institute of Management Science and Engineering, Henan University, Kaifeng 475004, China; 2 School of Business, Henan University, Kaifeng 475004, China; 3 School of Management & Economics, North China University of Water Resources and Electric Power, Zhengzhou 450046, China

**Keywords:** price game, carbon emission reduction, fairness concern, complexity

## Abstract

This paper studies a low-carbon dual-channel supply chain in which a manufacturer sells products through the direct channel and traditional channel, and a retailer sells products through the traditional channel. The manufacturer considers carbon emission reduction and has fairness concern behavior. The retailer provides sales service in the traditional channel and considers fairness concern behavior. The objective of this paper is to analyze the effects of different parameter values on the price stability and utility of the supply chain system emphatically using 2D bifurcation diagram, parameter plot basin, the basins of attraction, chaos attractor and sensitivity to the initial value, etc. The results find that the retailer’s fairness concern behavior shrinks the stability of the supply chain system more than that of the manufacturer’s fairness concern behavior. The system stability region decreases with the increase of carbon emission reduction level and the retailer’s fairness concern. The customers’ preference for the direct channel decreases the stable range of the direct channel, while it enlarges the stable range of the traditional channel. The supply chain system enters into chaos through flip bifurcation with the increase of price adjustment speed. In a stable state, the manufacture improving customer’s preference for the direct channel and the retailer choosing the appropriate fairness concern level can achieve the maximum utility separately. In a chaotic state, the average utilities of the manufacturer and retailer all decline, while that of the retailer declines even more. By selecting appropriate control parameter, the low-carbon dual-channel supply chain system can return to a stable state from chaos again. The research of this paper is of great significance to price decisions of participants and supply chain operation management.

## 1. Introduction

Under the enormous environmental pressure, the low-carbon economy based on low energy consumption [1], low emission and low pollution has gradually become the focus of global attention. For example, Coca-Cola has built a low-carbon supply chain to achieve a win-win situation of environmental protection and economy. Most production, sales, and consumers of Coca-Cola’s beverages are finished in the same area. By reducing air travel and introducing biodiesel technology, distribution fleets can solve many of the fuel problems. Low-carbon economic model poses new challenges to supply chain management, and the exploration of theory and practice of low-carbon supply chain management has become an urgent issue of the times. Scholars have extensively studied the contract, and optimal operation of the supply chain based on low-carbon environment [2,3]. Hui et al. [4] studied a low-carbon closed-loop supply chain in five scenarios to analyze the optimal operation in the competitive pricing and the carbon emission reduction (CER). Xia et al. [5] demonstrated that reciprocal preferences and CER significantly affected decision making and the system’s efficiency. Zhang et al. [6] studied the influence of the retailer’s fairness concern and government subsidies on optimal solutions of the system. Cicconi et al. [7] studied lightweight engineering, which is a current topic in mechanical industry, the results show the possibility to achieve about 20% of mass reduction for one casting, the related reduction in terms of carbon emission is about 7%. Considering the manufacturer’s overconfidence and consumer’s low-carbon preference, Ji et al. [8] analyzed the joint decisions on carbon emission reduction and inventory replenishment, and surprisingly found regulation parameters affect low-carbon operations. Du et al. [9] developed a Stackelberg model to game-theoretically analyze the decentralized decisions considering low-carbon effort of the manufacturer and retailer. Ji et al. [10] developed a Stackelberg game model and focused on the emission reduction in a dual-channel supply chain. The scholar found that the more profitable way for the manufacturer and retailer is the joint emission reduction strategy. Zhou et al. [11] developed two contracts considering advertising and emission reduction to optimize the decision and coordination of low-carbon supply chain when the retailer had fairness concerns. Zhou and Ye [12] developed a differential game model in a dual-channel supply chain considering joint emission reduction strategies. Rahmani and Mahoodian [13] studied the reliability and robustness of a supply chain network considering the uncertain demand and production cost. Qin et al. [14] adopted three demand forecasting scenarios to study pricing strategies and carbon emission reduction level. Scholars also expanded the environment view to supply chain operations using carbon regulations [15] and carbon tax [16]. 

The above literature studied the price decisions of low-carbon supply chain considering CER and customer’s preference for low-carbon products. However, in what range can the decision-maker adjust the parameters to make the supply chain system stable? What factors will affect customers’ channel choice and decision-makers’ profits?

In general, the consumer’s channel choice and profit in a supply chain can be affected by service input of decision-maker. Scholars have studied the impact of service input on price decision-making and stability of a dual-channel closed-loop supply chain system [17,18,19]. Ma et al. [20] developed a dual-channel game model considering the input of retailing service, analyzed the effect of service input adjustment speed on the system complexity and market performance. Jafar et al. [21] studied a Stackelberg game model in which decision-makers competed on price and service in a simple price discount contract simultaneously, they found that offering discount by manufacturer and providing the service by the retailer is an effective tool for the coordination of supply chain. Zhou et al. [22] investigated the effect of free-riding on the two members’ service strategies and profits under two scenarios, found the retailer preferred the non-differential pricing scenario and the manufacturer preferred a differential pricing scenario. Zhang and Wang [23] developed two dynamic pricing models and put forward a two-part tariff contract, focused on the influence of service value on the decisions and the complexity of two models. Dan et al. [24] examined the manufacturer’s warranty service decision and the value-added service competition in a dual-channel supply chain. They found that the higher the warranty service level was, the weaker the value-added service competition was, and there was no value-added service competition when the warranty service level was high enough. 

Fairness concern is another important factor affecting decision-makers’ profits and should be paid attention to by enterprises and scholars [25,26]. Li et al. [27] studied a two-echelon supply chain for the price and CER decisions with a fairness-concerned retailer, and found that, with higher emission reduction cost, the retailer was more concerned about fairness. Ma et al. [28] investigated closed-loop supply chains under four-channel structures to provide the optimal marketing effort, collection rate and pricing decisions for participators of the supply chain. Chen et al. [29] investigated pricing decision and ordering decision in a dyadic supply chain with buyback guarantee financing and fairness concerns, studied how the fairness concern and buyback guarantee financing affected participants’ equilibrium strategies and supply chain performance. Li and Li [30] considered the Stackelberg game model considering the retailer exhibits fairness concerns or not, found that channel efficiency grew with customer loyalty to the retail channel and fell with increasing in the retailer’s fairness concerns. Niu et al. [31] analyzed the supplier’s decision whether to open an online direct channel or not incorporating channel power and fairness concern, found that the supplier’s fairness concern effectively reduces his or her incentives to open an online channel. Liu et al. [32] analyzed the impacts of the fairness concerns on production sustainability level, low-carbon promotion level and profitability of supply chain, but they did not consider the dual-channel structure and channel service. Du et al. [33] indicated that fairness concerns could promote and coordinate the supplier and manufacturer to invest more in the sustainable development of green technology innovation. Li et al. [34] found the market shares of retailers are related to the impact of manufacturer’s fairness concern on retailers’ profits. Ma et al. [28] studied the pricing decisions of a closed-loop supply chain considering market effort and fairness concern. Liang and Qin [35] developed an estimation game model by fuzzy theory with fuzzy fairness concern. 

From the above literature, we can see that in different scenarios, the fairness concerns of decision-makers have a certain impact on the pricing, profit and coordination of supply chain products. How the fairness concerns of decision-makers affect the dynamic evolution process of the supply chain system is a hot topic worth studying.

The supply chain system exhibits complex dynamic characteristics in the process of evolution. Puu [36] briefly analyzed three oligopoly competition conditions and pointed out strange attractors could appear in the duopoly model. Lou and Ma [37] studied the bifurcation and cyclic attractors of a two-parallel model in the supply chain, suggested that the price adjustment would affect the stability and profits. Wu and Ma [38] found the game model entered chaos in two ways: Flip fluctuation and Neimark–Sacker bifurcation. Huang and Li [39] found the probabilistic selling supply chain system would enter chaos with higher system’s entropy through flip bifurcation or Neimark–Sacker bifurcation. Ma and Wang [40] found that the stability of a closed-loop supply chain is influenced by the retailer’s competitive position. Li and Ma [41] analyzed the system stability affected by the customers’ risk aversion and preference for a probabilistic product. Huang et al. [42] investigated the influences of parameters on the stability of three dynamic game models with the risk-averse manufacturer providing a complementary product. Matouk et al. [43] analyzed the stability of discrete-time dynamical game model and discussed the Neimark–Sacker bifurcation. The papers mentioned above provided the methods and perspectives by analyzing the complex characteristics of the supply chain. 

The low-carbon economy has gradually become the focus of global attention, it is a very interesting topic to study the stability and complex characteristics of the low-carbon dual-channel supply chain system affected by fairness concern behavior, retailer service and carbon emission reduction.

In this paper, we consider a low-carbon dual-channel supply chain which includes a fairness concern manufacturer providing low-carbon products and a fairness concern retailer providing sales service, and develop a dynamic vertical Nash game model in which the manufacturer sells the low-carbon products through the traditional channel and direct channel, the retailer sells products through the traditional channel. Using game theory and dynamic theory, the complex dynamic behaviors of the low-carbon dual-channel supply system under different parameter values are explored.

The paper is organized as follows: In Section 2, the model description is described. Equilibrium points and local stability of the dynamic system are shown in Section 3. In Section 4, the dynamic characteristics and utility of the dynamic system with parameters changing are presented. We give global stability analysis of the dynamic system in Section 5. Chaos control of the dynamic system is adopted in Section 6. Conclusions are drawn in Section 7.

## 2. Model Description

### 2.1. Basic Model Description

**Figure 1:**
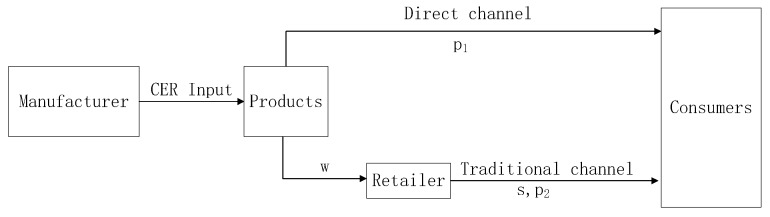
The low-carbon dual-channel supply chain system.

In this paper, we study a low-carbon dual-channel supply chain in which a manufacturer has fairness concern behavior and low carbon behavior, sells low-carbon products through the direct selling channel and traditional channel. A retailer has fairness concern behavior and provides sales service, and sells low-carbon products through the traditional channel (as shown in Figure 1). The manufacturer and retailer make the price games in the low-carbon dual-channel supply chain in order to obtain maximum profits.

### 2.2. Symbol Description

The symbols used in this paper are shown as follows:


$p_{i},\;i=1,2$
Retail price of products in the direct channel and traditional channel
k
The level of carbon emission reduction of the manufacturer
a
The basic market scale of products
w
Wholesale price of products
c
Production cost of products
s
The service input of the traditional channel provided by the retailer
$b_{i}$
The price elasticity coefficient of price (i=1,2)
$c_{i}$
The cross-price elasticity coefficient of price (i=1,2)
$D_{i},\;i=m,r$
Demand for products
$C_{k}$
The cost of carbon emission reduction of products
$C_{s}$
The service cost of the retailer
θ∈(0, 1)
The customer’s loyalty to the direct channel
β∈(0, 1)
The customer’s loyalty to the demand caused by carbon emission reduction
d
The sensitivity of the demand for carbon emission reduction
ε
Cost coefficient of carbon emission reduction of products
m
Cost coefficient of service
$\pi_{i},\;i=m,\;r$
The profits of the manufacturer and retailer
$U_{i},\;i=m,\;r$
The utilities of the manufacturer and retailer

### 2.3. Profit Functions

Market demand is affected by product price, service level, and CER level. According to the literature [4,10,11], assuming that the market demand is a linear function of product price, return rates, service level, and CER level:

(1) $$\{\begin{array}{l} D_{m}=\theta a-b_{1}p_{1}+c_{1}\left(p_{2}-s\right)+\beta dk\\ D_{r}=\left(1-\theta \right)a-b_{2}\left(p_{2}-s\right)+c_{2}p_{1}+\left(1-\beta \right)dk \end{array}$$

where θa expresses the customer number preferring the direct channel, while (1−θ)a represents the number of customers who prefer the traditional channel of the retailer. Formula 1 shows that customer demand is negatively correlated with channel product price and positively correlated with channel service and CER level.

The costs of CER and service are as follows [9,23]:

(2) $$\{\begin{array}{c} C_{k}=\frac{\varepsilon k^{2}}{2}\\ C_{s}=\frac{ms^{2}}{2} \end{array}$$

The profits of the manufacturer and retailer are as follows:

(3) $$\{\begin{array}{l} \pi_{m}=\left(p_{1}-c\right)D_{m}+\left(w-c\right)D_{r}-\frac{\varepsilon k^{2}}{2}\\ \pi_{r}=\left(p_{2}-w\right)D_{r}-\frac{ms^{2}}{2} \end{array}$$

Let $\pi_{mt}=\left(w-c\right)D_{r}$ be the manufacturer’s profit obtained from the traditional channel.

In market competition, the decision-makers show fairness concern behavior, which concerns not only their own profit, but also the competitor’ profit. Inspired by Du et al. [44] and Cui et al. [45], the utility functions of the manufacturer and retailer can be constructed as follows:

(4) $$\{\begin{array}{l} U_{m}=\pi_{m}-\lambda_{2}\left(\pi_{r}-\gamma \pi_{mt}\right)\\ \;U_{r}=\pi_{r}-\lambda_{1}\left(\pi_{mt}-\pi_{r}\right) \end{array}$$

where $\lambda_{1},\;\;\lambda_{2}\in \left(0,\;1\right)$ represent the fairness concern coefficient of the manufacturer and retailer. γ∈(0,1) denotes the relative profit coefficient, the retailer takes the absolute profit comparing with the manufacturer’s profit in the direct channel. $\pi_{r}-\gamma \pi_{mt}$ means the manufacturer only takes part of the profit comparing with the retailer’s profit in the traditional channel. On one hand, only if $\pi_{r}<\pi_{mt}$, the retailer’s utility will decrease, on the other hand, only if $\pi_{r}>\gamma \pi_{mt}$, the utility of manufacturer will decrease.

According to the above equations, taking the first-order partial derivatives of $U_{m}$ and $U_{r}$ on $p_{1}$ and $p_{2}$ respectively, the marginal utilities of the manufacturer and retailer can be obtained.

$$\frac{\partial U_{m}}{\partial p_{1}}=dk\beta +a\theta -b_{1}p_{1}+\left(w-c\right)c_{2}+b_{1}\left(c-p_{1}\right)+c_{1}\left(p_{2}-s\right)+\gamma c_{2}\lambda_{2}\left(w-c\right)+c_{2}\lambda_{2}\left(w-p_{2}\right).\;\frac{\partial U_{r}}{\partial p_{2}}=c_{2}p_{1}-dk\left(1-\beta \right)+a\left(1-\theta \right)+b_{2}\left(s-p_{2}\right)+b_{2}\left(w-p_{2}\right)+\lambda_{1}dk\left(1-\beta \right)+\;a\left(1-\theta \right)+b_{2}\left(w-c\right)+c_{2}p_{1}+b_{2}\left(s-p_{2}\right)+b_{2}\left(w-p_{2}\right)$$

Solving $\frac{\partial U_{m}}{\partial p_{1}}=0,\;\;\frac{\partial U_{r}}{\partial p_{2}}=0,$ we can obtain the best decisions of the manufacturer and retailer:

(5) $$\{\begin{array}{ll} p_{1}^{*}= & \frac{dk\beta +a\theta +cb_{1}-sc_{1}-c\gamma c_{2}\lambda_{2}+\left(-c+w\right)c_{2}+wc_{2}\lambda_{2}\left(1+\gamma \right)}{2b_{1}}+\frac{2b_{1}\left(c_{1}-c_{2}\lambda_{2}\right)\left[sb_{2}+wb_{2}+b_{2}\lambda_{1}\left(s-c+2w\right)-dk\left(\beta -1\right)\left(1+\lambda_{1}\right)\right]}{8b_{2}b_{1}^{2}\left(1+\lambda_{1}\right)-c_{2}2b_{1}\left(1+\lambda_{1}\right)\left(c_{1}-c_{2}\lambda_{2}\right)}\\ & +\frac{2ab_{1}\left(c_{1}-c_{2}\lambda_{2}\right)\left(1-\theta \right)\left(1+\lambda_{1}\right)}{8b_{2}b_{1}^{2}\left(1+\lambda_{1}\right)-c_{2}2b_{1}\left(1+\lambda_{1}\right)\left(c_{1}-c_{2}\lambda_{2}\right)}+\frac{c_{2}\left(1+\lambda_{1}\right)\left(c_{1}-c_{2}\lambda_{2}\right)\left[dk\beta +a\theta +cb_{1}-sc_{1}+c_{2}\left(-c+w\right)-c\gamma c_{2}\lambda_{2}+wc_{2}\lambda_{2}\left(1+\gamma \right)\right]}{8b_{2}b_{1}^{2}\left(1+\lambda_{1}\right)-c_{2}2b_{1}\left(1+\lambda_{1}\right)\left(c_{1}-c_{2}\lambda_{2}\right)}\\ p_{2}^{*}= & \frac{2b_{1}\left(\left(1+\lambda_{1}\right)\left(dk\left(1-\beta \right)+a\left(1-\theta \right)\right)+sb_{2}+wb_{2}+b_{2}\lambda_{1}\left(-c+s+2w\right)\right)}{\left(1+\lambda_{1}\right)\left[4b_{1}b_{2}+c_{2}\left(-c_{1}+c_{2}\lambda_{2}\right)\right]}+\frac{c_{2}\left(1+\lambda_{1}\right)\left[dk\beta +a\theta +cb_{1}-sc_{1}+c_{2}\left(-c+w\right)-c\gamma c_{2}\lambda_{2}+wc_{2}\lambda_{2}\left(1+\gamma \right)\right]}{\left(1+\lambda_{1}\right)\left[4b_{1}b_{2}+c_{2}\left(-c_{1}+c_{2}\lambda_{2}\right)\right]} \end{array}$$

As a matter of fact, when players make price decisions, they cannot get all the information of competitors and adjust their price strategy with bounded rationality. The manufacturer and retailer will make their price decision in next period based on the marginal utility of the current period. When the marginal utility of the current period is positive, the manufacturer and retailer will raise the price adjustment speed in the next period, otherwise, reduced in the next period. A discrete dynamic dual-channel Nash game model can be described:

(6) $$\{\begin{array}{c} p_{1}\left(t+1\right)=p_{1}\left(t\right)+\alpha_{1}p_{1}\left(t\right)\frac{\partial U_{m}\left(t\right)}{\partial p_{1}\left(t\right)}\\ p_{2}\left(t+1\right)=p_{2}\left(t\right)+\alpha_{2}p_{2}\left(t\right)\frac{\partial U_{r}\left(t\right)}{\partial p_{2}\left(t\right)} \end{array}$$

where $\alpha_{1},\alpha_{2}$ represent the price adjustment speeds of the low-carbon dual-channel supply chain.

## 3. Equilibrium Points and Local Stability of Dynamic System (6)

### 3.1. Equilibrium Points

According to the dynamic system (6), the four equilibrium solutions can be obtained by making $p_{1}\left(t+1\right)=p_{1}\left(t\right),\;\;p_{2}\left(t+1\right)=p_{2}\left(t\right)$:

$$\begin{array}{l} E_{1}=\left(0,\;0\right)\\ E_{2}=\left(\frac{dk\beta +a\theta +cb_{1}-sc_{1}+c_{2}\left(w-c\right)+c_{2}\lambda_{2}\left(w-c\gamma +w\gamma \right)}{2b_{1}},\;0\right)\\ E_{3}=\left(0,\;\;\frac{a\left(1-\theta \right)+dk\left(1-\beta \right)+b_{2}\left(s+w\right)+\lambda_{1}\left(a+dk-dk\beta -a\theta -cb_{2}+sb_{2}+2wb_{2}\right)}{2b_{2}\left(1+\lambda_{1}\right)}\right)\\ E_{4}=\left(p_{1}^{*},\;p_{2}^{*}\right) \end{array}$$

In order to analyze the stability of equilibrium points, the Jacobian matrix of the dynamic system (6) is given as follows:

(7) $$J\left(p_{1},\;p_{2}\right)=\left|\begin{array}{cc} 1+\alpha_{1}f_{1} & \alpha_{1}\left(c_{1}-c_{2}\lambda_{2}\right)p_{1}\\ \alpha_{2}c_{2}\left(1+\lambda_{1}\right)p_{2} & 1+\alpha_{2}f_{2} \end{array}\right|\;\;\;\;\;\;\;\;\;\;\;\;\;\;$$

where

$$f_{1}=-2b_{1}p_{1}+dk\beta +a\theta +\left(-c+w\right)c_{2}+b_{1}\left(c-p_{1}\right)-b_{1}p_{1}+c_{1}\left(-s+p_{2}\right)-c_{2\lambda_{2}}c\gamma -w\left(1+\gamma \right)+p_{2},\;\;f_{2}=-2b_{2}p_{2}\left(1+\lambda_{1}\right)+\left(1+\lambda_{1}\right)-dk\left(-1+\beta \right)-a\left(-1+\theta \right)+c_{2}p_{1}+b_{2}s+w+\left(-c+s+2w\right)\lambda_{1}-2p_{2}\left(1+\lambda_{1}\right)$$

The stability of the equilibrium points is determined by the eigenvalues of the Jacobian matrix evaluated at the corresponding equilibrium points. According to Routh–Hurwitz condition, the necessary and sufficient conditions for the stability of the equilibrium point of the system are that all the non-zero eigenvalues are greater than one.

Substituting $E_{1},\;E_{2},\;E_{3}$ into the Jacobian matrix (7), we can get the Proposition 1.

**Proposition** **1.** $E_{1},\;E_{2},\;E_{3}$*are boundary equilibrium points and unstable points*.

**Proof.**  For equilibrium point $E_{1}$, the Jacobian matrix of the dynamic system (6) is equal to

$$J\left(E_{1}\right)=\left|\begin{array}{cc} 1+\alpha_{1}A_{1} & 0\\ 0 & 1+\alpha_{2}A_{2} \end{array}\right|,$$

where

$$\begin{array}{l} A_{1}=dk\beta +a\theta +cb_{1}-sc_{1}+c_{2}\left(w-c\right)+c_{2}\lambda_{2}\left[w+\gamma \left(w-c\right)\right],\\ A_{2}=\left(1+\lambda_{1}\right)dk\left(1-\beta \right)+a\left(1-\theta \right)+\alpha_{2}b_{2}s+w+\lambda_{1}\left(s+2w-c\right). \end{array}$$

its two eigenvalues are as follows:

$$\begin{array}{l} r_{1}=1+\alpha_{1}\left\{dk\beta +a\theta +cb_{1}-sc_{1}+c_{2}\left(w-c\right)+c_{2}\lambda_{2}\left[w+\gamma \left(w-c\right)\right]\right\},\\ r_{2}=1+\alpha_{2}\left[\left(1+\lambda_{1}\right)dk\left(1-\beta \right)+a\left(1-\theta \right)+\alpha_{2}b_{2}s+w+\lambda_{1}\left(s+2w-c\right)\right]. \end{array}$$

Obviously, $r_{1\;}>1,\;\;r_{2}>1$, which implies that $E_{1}$ is unstable. We can prove $E_{2}\;\mathrm{and}\;E_{3}$ are all unstable equilibrium points in the same way. □

The following analyzes the stability of the Nash equilibrium point ($E_{4}$), the expression of the characteristic polynomial of the Jacobi matrix in $E_{4}$ can be expressed as:

(8) $$f\left(\lambda \right)=\lambda^{2}+A\lambda +B$$

where $A=2+\alpha_{1}f_{1}+\alpha_{2}f_{2}$, B= ($1+\alpha_{1}f_{1})\;$($\;1+\alpha_{2}f_{2})-\alpha_{1}\left(c_{1}-c_{2}\lambda_{2}\right)p_{1}\;\alpha_{2}c_{2}\left(1+\lambda_{1}\right)p_{2}$.

A and B represent the trace and determinant of the Jacobian matrix J($p_{1}^{*},\;p_{2}^{*})$, respectively.

As for $E_{4\;}$, the necessary and sufficient condition of asymptotic stability is that all the eigenvalues are inside the unit circle in a complex plane. The stable conditions must satisfy the following conditions:

(9) $$\{\begin{array}{l} F\left(1\right)=1-A+B>0\\ F\left(-1\right)=1+A+B>0\\ F\left(0\right)=1-\left|B\right|>0 \end{array}$$

By solving condition (9), the stability domain of the dynamic system (6) can be obtained. Due to these limitations being so complex, solving the inequality Equation (9) is very complicated. If $E_{4\;}$ satisfies the inequality Equation (9), we may ensure that the dynamic system (6) is locally stable. Next, we give the stable region of the dynamic system (6) through numerical simulation. 

According to the current situation and characteristics of the dual-channel supply chain enterprises and referring to the parameter values in the literature [37,39], setting parameter values as follows: $a=100,\;w=10,\;\;\theta =0.6,\;b_{1}=3,b_{2}=2,c_{1}=c_{2}=0.5,d=5,k=12,s=2,\varepsilon =5,m=0.6,\beta =0.5,\;\;c=6,\;\;\lambda_{1}=0.2,\;\lambda_{2}=0.2,\;\gamma =0.4$. 

According to the parameter values above, we can obtain $E_{4}=\left(37.69,\;30.87\right)$.

The Jacobian matrix is

(10) $$J\left(E_{4}\right)=\left|\begin{array}{cc} 1-185.24a_{1} & 12.35a_{1}\\ 22.62a_{2} & 1-180.92a_{2} \end{array}\right|$$

The characteristic equation of the Jacobian matrix (10) is

(11) $$f\left(\lambda \right)=\lambda^{2}+A\lambda +B,$$

where $A=2-185.24a_{1}-180.92a_{2}$, $B=\left(1-185.24a_{1}\right)\left(1-180.92a_{2}\right)-279.28a_{1}a_{2}$. 

**Figure 2:**
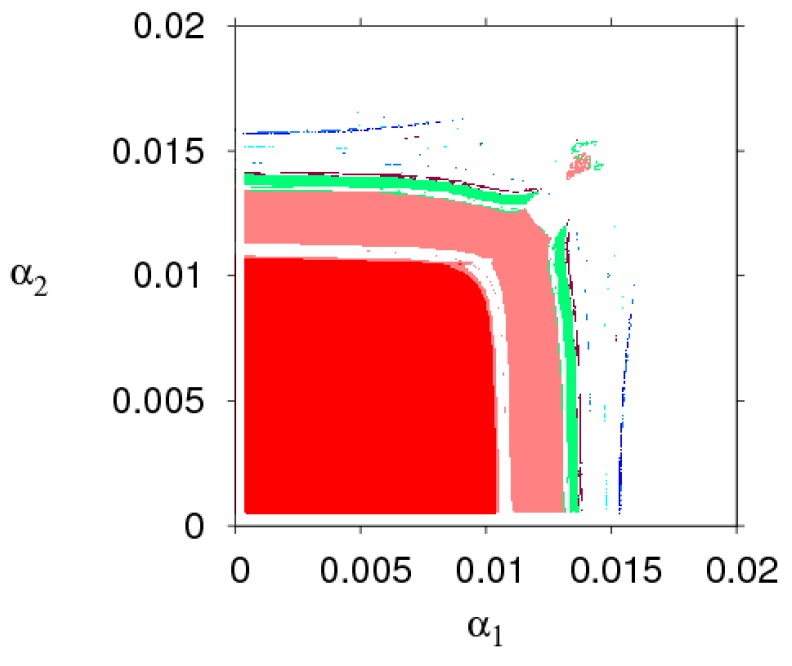
The evolution process of dynamic system (6) in the ($\alpha_{1},\;\alpha_{2}$) plane.

The 2D bifurcation diagram (the parameter basin) is a powerful tool for numerical simulation. Next, we will use the parameter basin to analyze the evolution process of the stability region of the dynamic system (6). Based on the stability conditions (9), the parameter basin of the dynamic system (6) is simulated in Figure 2, which shows the route of the dynamic system (6) to chaos. Different periods are represented by different colors, stable (red), period-2 (pink), period-3 (yellow), period-4 (light blue), period-5 (blue), period-7 (orange), period-8 (Crimson), chaos and divergence (white). The dynamic system (6) goes into chaos through periodic doubling bifurcation with $\alpha_{1}\;\mathrm{or}\;\alpha_{2}$ increasing.

### 3.2. The Stable Region of the Dynamic System (6) for Parameters Changing

**Figure 3:**
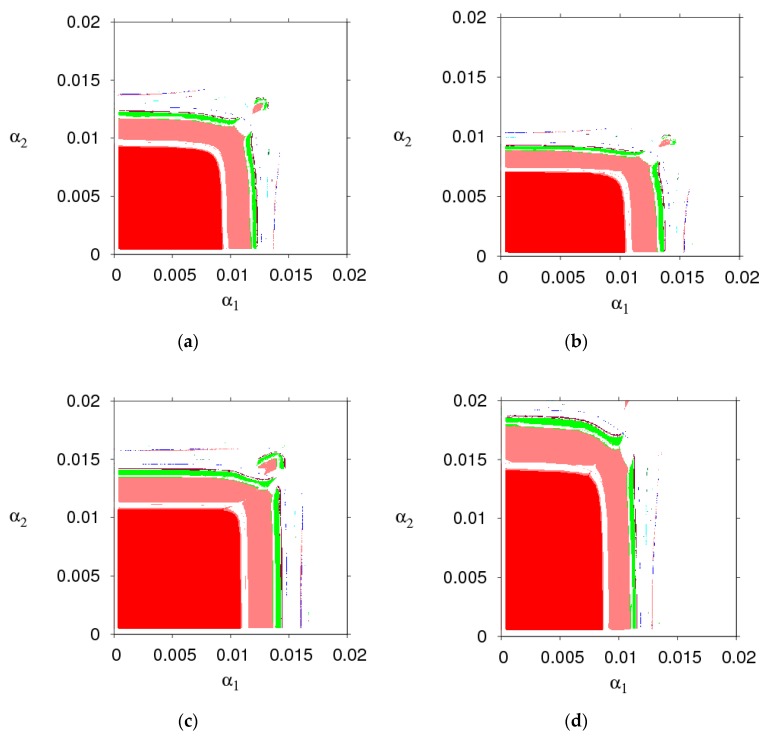
The evolution process of the dynamic system (6) with different parameters values. (**a**) k=20, (**b**) $\lambda_{1}=0.8$, (**c**) $\lambda_{2}=0.8$, (**d**) θ=0.8.

Figure 3 shows the changes in the stability region of the dynamic system (6) when $k,\;\lambda_{1},\;\lambda_{2}$ and θ take different values. By comparing the size of the stability region (red region) in Figure 3a,d, we can find the high level of fairness concern of the retailer will shrink the stability region of Nash equilibrium point, while fairness concern level of manufacturer has little effect on system’s stability. Increasing the CER of the manufacturer will decrease the stable region of dynamic system (6). The increasing number of customers preferring the online channel will make the stable region of the manufacturer decrease and that of the retailer increase. Therefore, the manufacturer and retailer should adjust the parameters according to the actual market conditions so that the dynamic system (6) is in a stable state.

**Figure 4:**
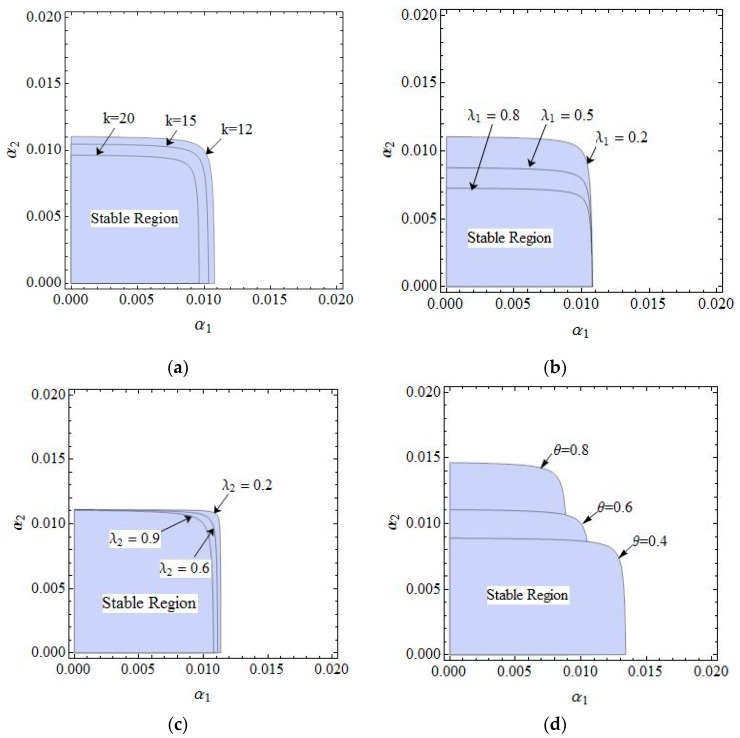
The stability regions in the phase plane of ($\alpha_{1},\;\alpha_{2})\;$ with different parameter values. (**a**) The stable region with k changing, (**b**) The stable region with $\lambda_{1}$ changing, (**c**) The stable region with $\lambda_{2}$ changing, (**d**) The stable region with θ changing.

Figure 4 gives a more intuitive analysis of the effect of parameter changes on the stability region of the dynamic system (6). Figure 4a shows the stability regions of the dynamic system (6) with the parameter k having different values when other parameters are fixed. We can see that the stability region is the biggest when k = 12, and becomes smaller gradually when k = 15, 20. Figure 4b shows the stability regions of the dynamic system (6) with different values of $\lambda_{1}$, the scope of $\alpha_{2}$ is decreasing and that of remains unchanged, the dynamic system (6) is more sensitive to $\alpha_{2}$ than $\alpha_{1}$ when $\lambda_{1}$ changes, so the retailer should be more cautious in adjusting the price strategy than the manufacturer. Similarly, the stability region of the dynamic system (6) remains unchanged with $\lambda_{2}$ increasing (showed in Figure 4c). The scope of $\alpha_{2}$ is increasing and that of $\alpha_{1}$ is decreasing with θ increasing (shown in Figure 4d), the stability of the dynamic system (6) is more sensitive to $\alpha_{1}$ than $\alpha_{2}$ with θ increasing.

From the above diagrams, some conclusions can be obtained: (1) The retailer’s fairness concern behavior has a greater impact on the stability of the dynamic system (6) than that of the manufacturer’s fairness concern behavior. (2) The manufacturer setting CER can shrink the stability region of the dynamic system (6), the customers’ preference for the direct channel decreases the stable range of price adjustment of the direct channel and enlarges the stable range of price adjustment of the traditional channel.

## 4. The Numerical Simulation of the Dynamic System (6)

### 4.1. The Complexity Entropy Analysis of the Dynamic System (6) with the Price Adjustment Speed 

**Figure 5:**
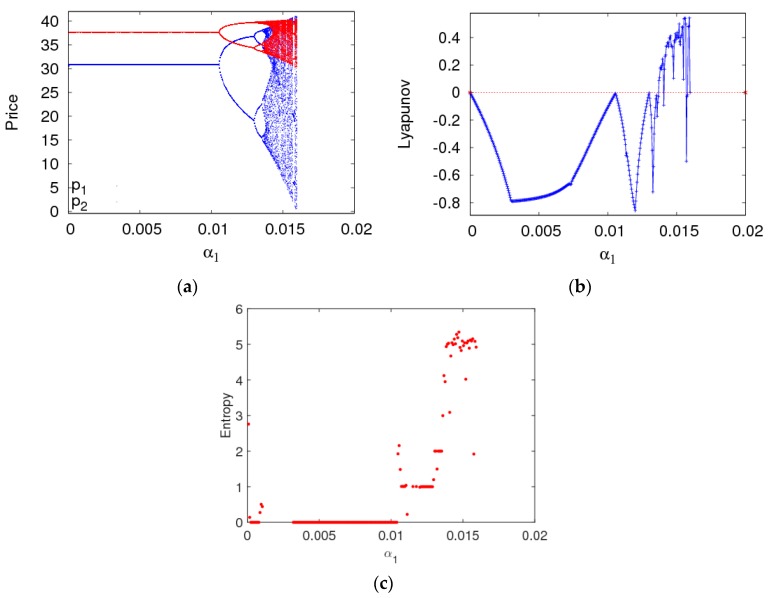
The behavior of dynamic system (6) with respect to $\alpha_{1}$ when $\alpha_{2}=0.008$. (**a**) The bifurcation diagram, (**b**) The LLE diagram, (**c**) The entropy diagram.

In this section, setting parameter values the same as the previous section, the dynamic behaviors and entropy of the dynamic system (6) are described with $a_{1}$ and $a_{2}$ varying. First of all, Figure 5a is a bifurcation diagram of the dynamic system (6) with the change of $a_{1}$ when $a_{2}=0.008$. The dynamic system (6) loses its stability through the flip bifurcation with $\alpha_{1}$ increasing from 0 to 0.011. The 2-period cycle appears with $\alpha_{1}$ increasing from 0.011 to 0.0131. The dynamic system (6) enters into a chaotic state finally through period doubling bifurcation. The LLE and entropy of the dynamic system (6) is shown in the Figure 5b,c with $\alpha_{1}$ varying from 0 to 0.016 when the dynamic system (6) is in a stable state, the LLE is negative and the entropy of the dynamic system (6) equals zero. When the dynamic system (6) is in a chaotic state, most of the LLEs are bigger than zero and the entropy of the dynamic system (6) is relatively large. Therefore, we can make a conclusion that the entropy of the dynamic system (6) will increase with the irrational change of price adjustment parameter, the manufacturer and retailer need to be very cautious about adjusting price parameters to keep the dynamic system (6) in a stable state.

**Figure 6:**
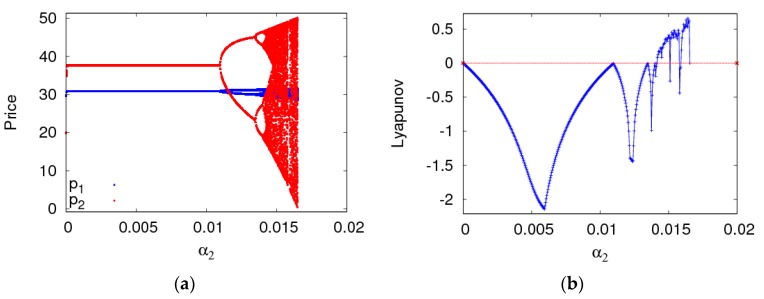

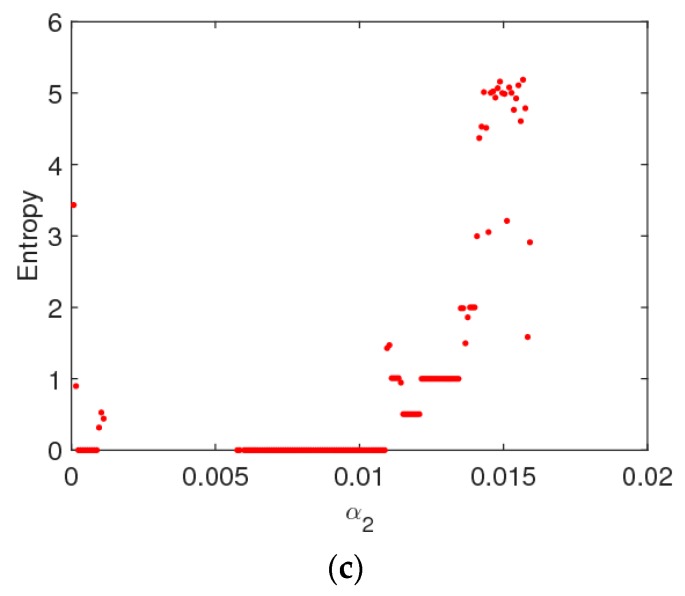
The behaviors of the dynamic system (6) with respect to $\alpha_{2}$ and $\alpha_{1}=0.005$. (**a**) The bifurcation diagram, (**b**) The LLE diagram, (**c**) The entropy diagram.

Figure 6 shows the retail price, the LLE and entropy diagram of the dynamic system (6) as $a_{2}$ changes when $a_{1}=0.005$. The dynamic system (6) goes into a chaotic state through flip bifurcation with $\alpha_{2}$ increasing. The LLEs of the dynamic system (6) are negative and the entropy of the dynamic system (6) equals zero when $\alpha_{2}<0.011$, most of the LLEs are bigger than zero and the entropy of the dynamic system (6) is relatively larger when $\alpha_{2}>0.0131$. 

Therefore, the instability and entropy of the dynamic system (6) will increase when the participants make irrational changes of price adjustment parameter, participants need to adjust price parameters very carefully in order to keep the dynamic system (6) stable.

**Figure 7:**
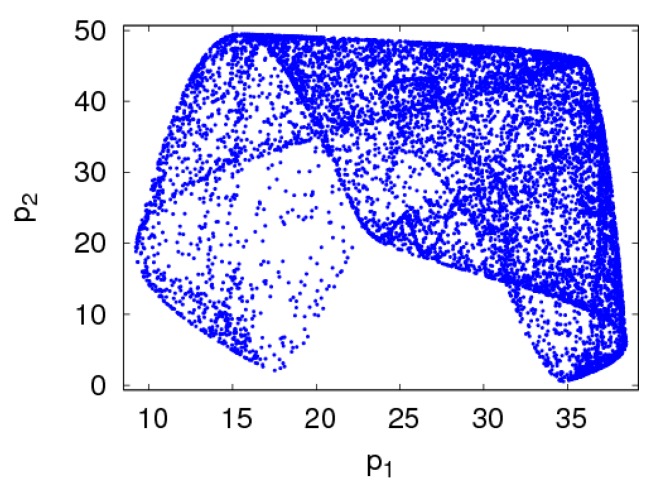
Chaos attractor of the dynamic system (6) with $\alpha_{1}=0.013,\alpha_{2}=0.015$.

The chaotic attractor of the dynamic system (6) is shown in Figure 7 when $\alpha_{1}=0.013,\alpha_{2}=0.015$. In the chaotic state, the retail prices of the manufacturer and retailer are in disorder. Chaotic attractor is an important feature to characterize the chaotic state of the dynamic system (6).

**Figure 8:**
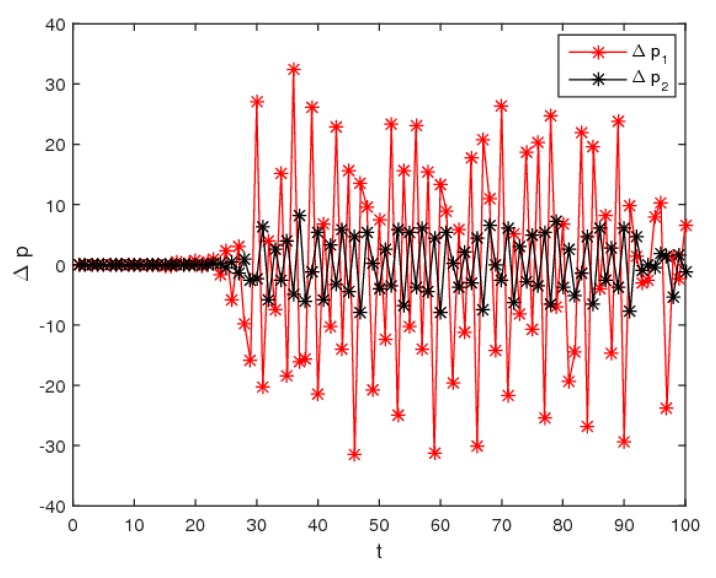
The sensitivity to initial value when $(p_{1},\;p_{2})=\;$ (28, 20) and (28.001, 20).

An important feature of the chaotic system is the sensitivity to initial values. Here, keeping $p_{2}$ unchanged, Figure 8 shows the differences of the evolution process of the dynamic system (6) when the initial value of $p_{1}$ only changes by 0.001. We can see that, the price values show no difference in the first 14 time iterations, but after that, the price shows a great difference. That is to say, the small difference of the initial value will cause a great deviation after much iteration, which provides us with the enlightenment that decision-makers should be more cautious in choosing the initial value of decision variables.

### 4.2. The Dynamic Characteristics of the Dynamic System (6) with Parameters Changing

In this section, the evolution characteristics of the dynamic system (6) are analyzed with the changes of θ,  ε,  β and $\lambda_{1}$ when the dynamic system (6) is in a stable state and period-2 state. By analyzing the influence of parameter changing on the system’s stability, decision-makers can make better decisions to keep the system in a stable competitive state.

**Figure 9:**
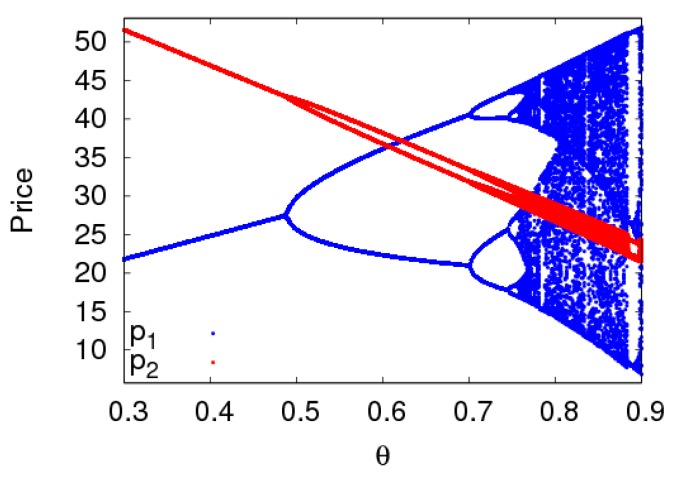
The bifurcation diagram of the dynamic system (6) with θ changing when $a_{1}=0.012$ and $a_{2}=0.005$.

The dynamic system (6) is in a period-2 state with $\alpha_{1}=0.012,\alpha_{2}=0.01$ when other parameters are fixed as above. Figure 9 shows the price bifurcation diagram of the dynamic system (6) with the change of θ. When θ≤0.484, the dynamic system (6) is in a stable state. When 0.484<θ≤0.694, the dynamic system (6) is in a period-2 state. When 0.749<θ≤0.9, the dynamic system (6) is in a chaotic state. We can see that when the dynamic system (6) is in a period-2 state, the adjustment of customers’ channel preference will bring the dynamic system (6) back to a stable state.

**Figure 10:**
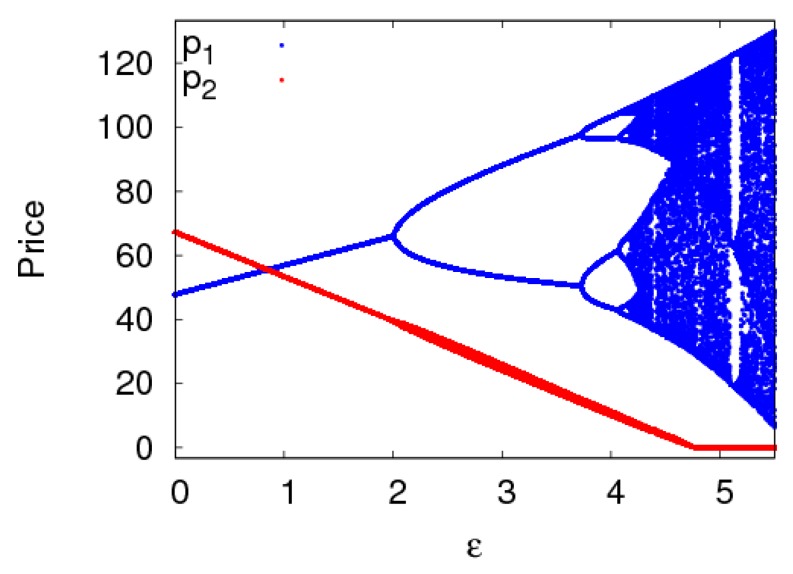
The bifurcation diagram of the dynamic system (6) with ε changing when $a_{1}=a_{2}=0.005$.

The dynamic system (6) is in a stable state with $\alpha_{1}=\alpha_{2}=0.005$ when other parameters are fixed as above. Figure 10 shows the evolutionary characteristics of the dynamic system (6) with change of ε. When ε≤2.15, the dynamic system (6) is in a stable state. When 2.15<θ≤3.75, the dynamic system (6) is in a period-2 state. When ε>4.2 the dynamic system (6) is in a chaotic state. In all, the cost coefficient of carbon emission reduction has a great impact on the stability of the dynamic system (6). In order to keep the system in a stable state, the manufacturer should pay attention to the size of cost coefficient of carbon emission reduction when making decisions.

**Figure 11:**
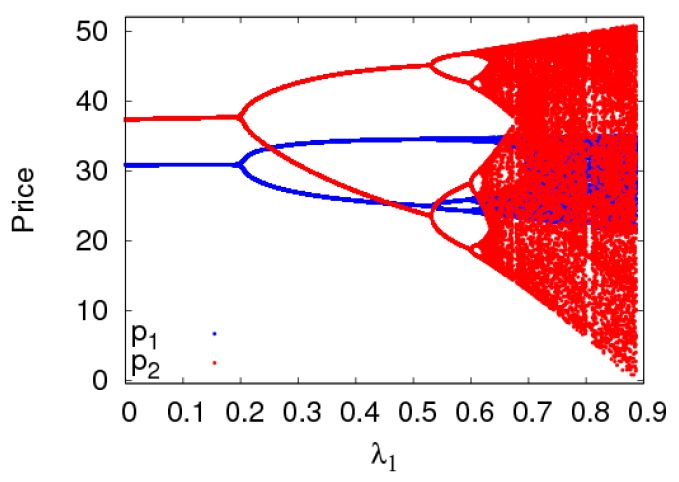
The bifurcation diagram of the dynamic system (6) with $\lambda_{1}$ changing when $a_{1}=0.01,a_{2}=0.01$.

The dynamic system (6) is in a stable state with $\alpha_{1}=\alpha_{2}=0.01$ when other parameters are fixed as above. Figure 11 shows the price evolution process of the dynamic system (6) with the change of $\lambda_{1}$. When $\lambda_{1}\leq 0.21$, the dynamic system (6) is in a stable state. When $0.21<\lambda_{1}\leq 0.62$, the dynamic system (6) is in a periodic doubling bifurcation state. When $\lambda_{1}>0.62$ the dynamic system (6) is in a chaotic state. Therefore, the dynamic system (6) can be in a stable state when the retailer’s fairness concern level is in a certain range when the adjustment parameters are fixed, otherwise the dynamic system (6) will enter a chaotic state.

**Figure 12:**
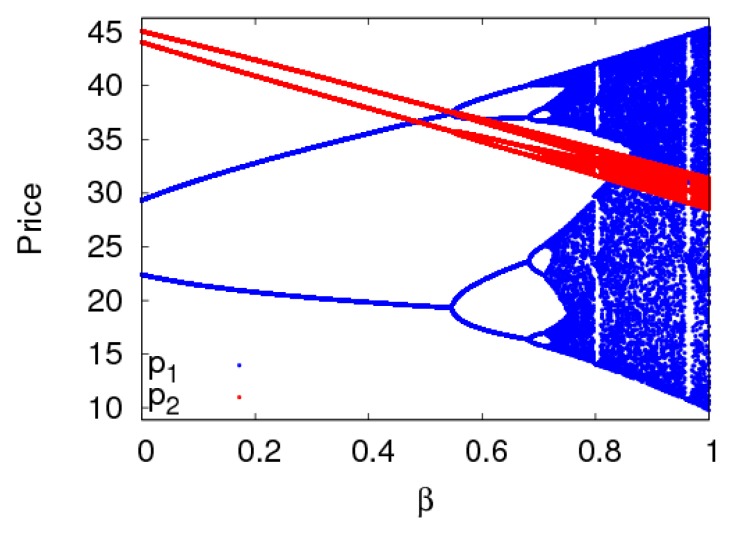
The bifurcation diagram of the dynamic system (6) with β changing when $a_{1}=0.013,a_{2}=0.008$.

The dynamic system (6) is in a period-2 state with $a_{1}=0.013,a_{2}=0.008$ when other parameters are fixed as above. Figure 12 shows the price evolution process of the dynamic system (6) with the change of β. When 0≤β≤0.68, the dynamic system (6) is in a periodic doubling bifurcation state. When β>0.68, the dynamic system (6) is in a chaotic state. Therefore, the dynamic system (6) can be in a periodic doubling state when the adjustment of customer channel preference caused by carbon emission reduction is in a certain range. Otherwise, the dynamic system (6) will enter a chaotic state.

### 4.3. The Effect of Price Adjustment Speed on the Utilities of the Dynamic System (6)

The change of parameter values will increase the instability of the dynamic system (6) obviously. It is complicated for the manufacturer and retailer to make dynamic price decisions in an unstable state. Therefore, we suspect that the utilities of two sides will also be influenced. This section mainly analyzes the influences of parameters changing on the average utilities of the manufacturer and retailer.

**Figure 13:**
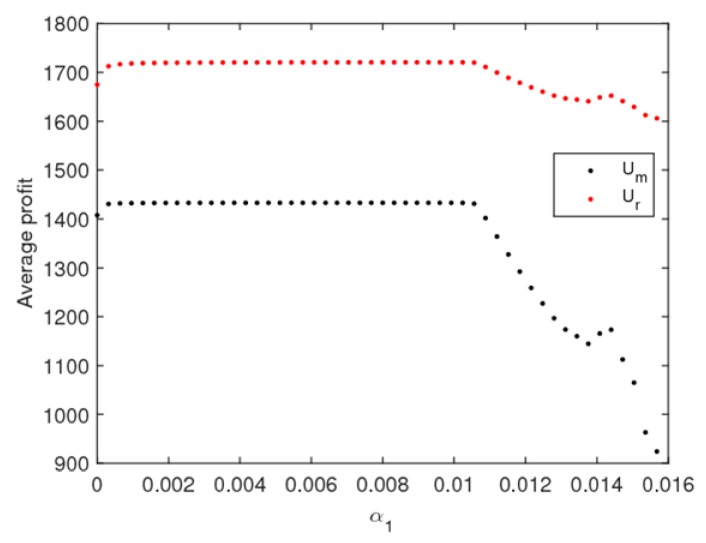
The average utilities of the manufacturer and retailer with $\alpha_{1}$ changing.

Figure 13 is the evolution diagram of the average utilities of the manufacturer and retailer with an increase of $\alpha_{1}$, we can see that, in the periodic doubling bifurcation and a chaotic state, the average utility of the manufacturer and retailer all decline, while that of the retailer declines even more.

**Figure 14:**
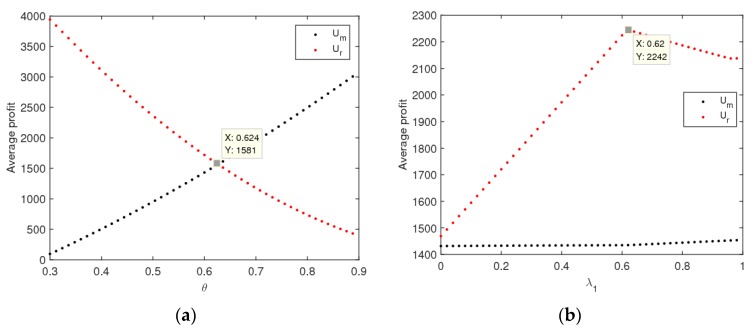
The effect of parameters changing on the utilities of the manufacturer and retailer with $\alpha_{1}=\alpha_{2}=0.008$. (**a**) θ, (**b**) $\lambda_{1}$.

Figure 14 describes the impact of θ and $\lambda_{1}$ on the utilities of the manufacturer and retailer when the dynamic system (6) is in a stable state. The utility of the manufacturer increases and that of the retailer decreases with θ increases, and the utility of the manufacturer is greater than that of the retailer when θ>0.624 (shown in Figure 14a). The utility of the retailer increases first and then decreases and reaches maximum values at $\lambda_{1}=0.62$. The utility of the manufacturer has hardly changed with $\lambda_{1}$ increases (shown in Figure 14b). Therefore, the manufacture should improve the customers’ preference for the direct channel and the retailer should choose the appropriate level of fairness concern to achieve the highest utility.

## 5. Global Stability of the Dynamic System (6)

The basins of attraction are an effective method for analyzing the influences of parameters changing on global stability, which includes attraction domain and escaping area. We make basins of attraction on the initial price $p_{1}$ and $p_{2}$ under different parameters values.

**Figure 15:**
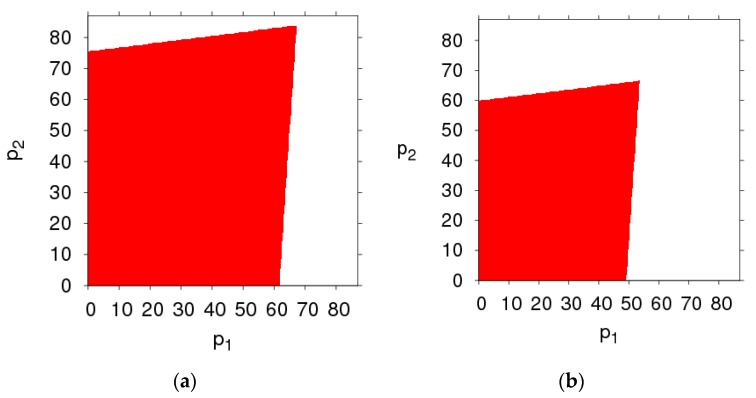

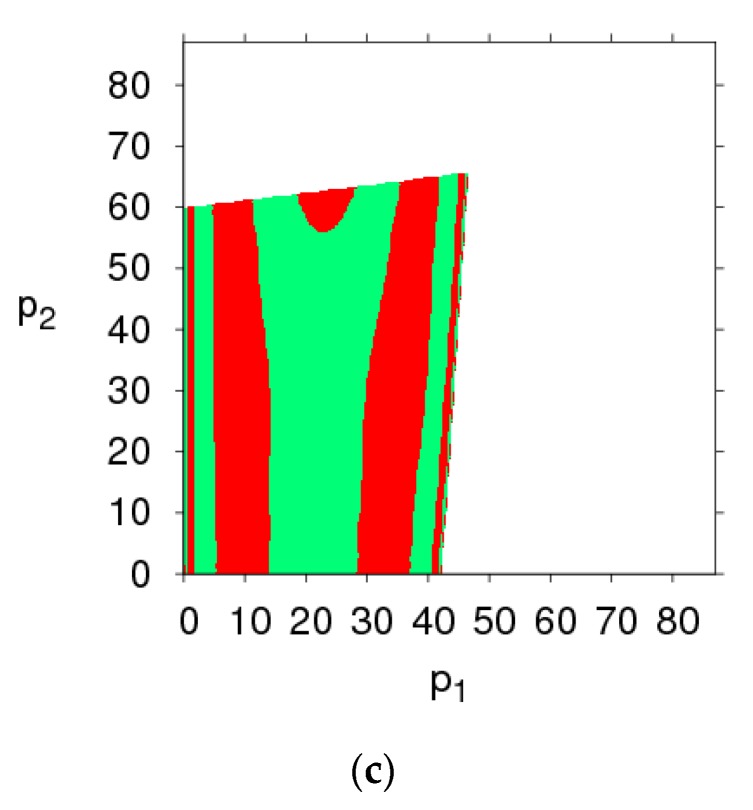
Basins of attraction of the dynamic system (6). (**a**) $\alpha_{1}=\alpha_{2}=0.005$, (**b**) $\alpha_{1}=\alpha_{2}=0.008,\;$ (**c**) $\alpha_{1}=0.012,\;\alpha_{2}=0.008$.

**Figure 16:**
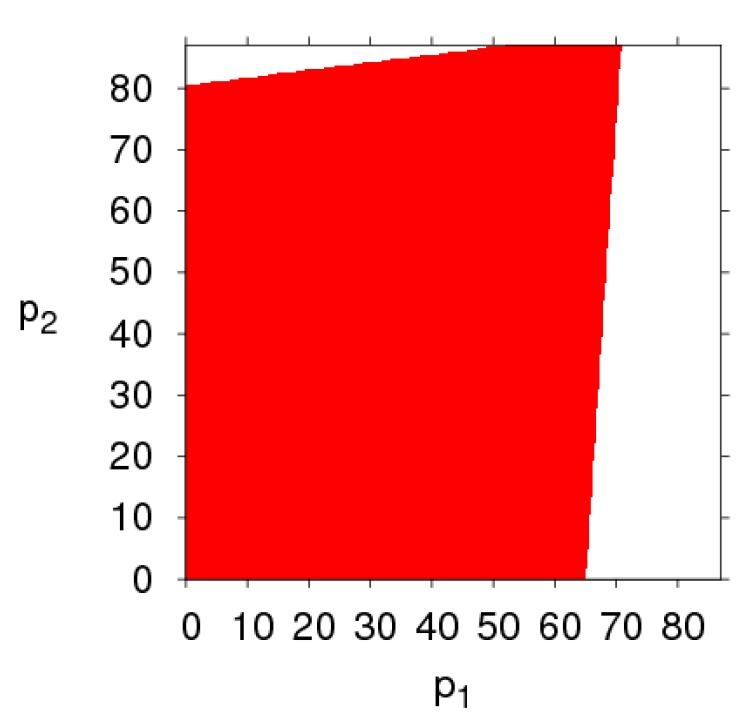
Basins of attraction of dynamic system (6), $\alpha_{1}=\alpha_{2}=0.005$, k=20.

**Figure 17:**
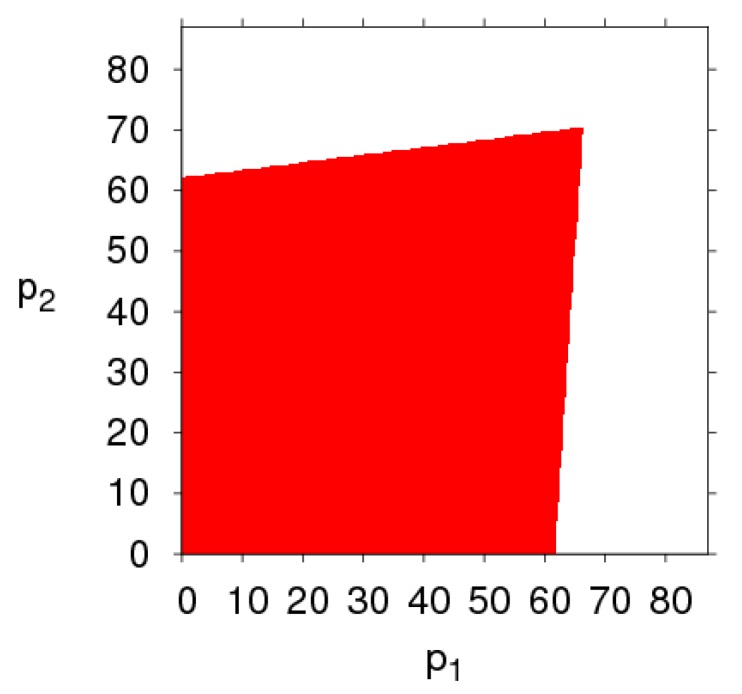
Basins of attraction of dynamic system (6), $\alpha_{1}=\alpha_{2}=0.005$, $\lambda_{1}=0.8$.

**Figure 18:**
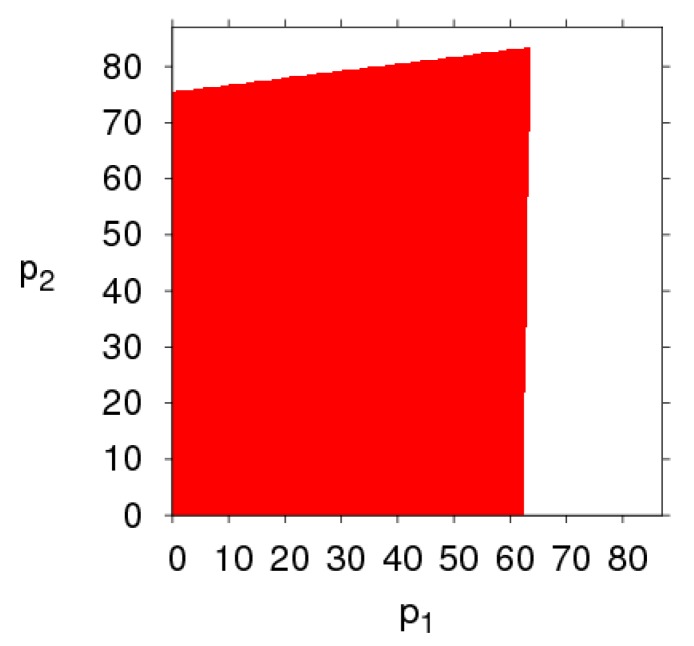
Basins of attraction of dynamic system (6), $\alpha_{1}=\alpha_{2}=0.005$, $\lambda_{2}=0.8$.

**Figure 19:**
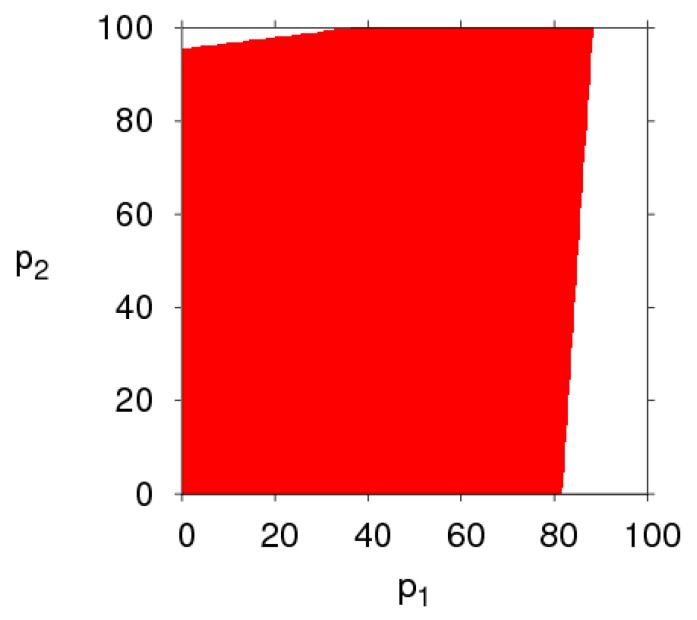
Basins of attraction of the dynamic system (6) when $\alpha_{1}=\alpha_{2}=0.005$ and a=400.

The basins of attraction are a set of initial conditions, if the initial price is taken from the attraction domain, the system will emerge the same attractor after a series of iterations. If the initial price is outside the basins of attraction, the system will fall into divergence at last. By fixing the parameter values of the dynamic system (6), as mentioned above, and setting different values for $\alpha_{1}$ and $\alpha_{2}$, respectively, the basins of attraction of $p_{1}$ and $p_{2}$ of the dynamic system (6) are shown in Figure 15 in which the red region denotes the stable attraction domain, light green denotes the period-2 attraction region and the white region denotes the escape area. Figure 16, Figure 17, Figure 18 and Figure 19 show the attraction regions of $p_{1}$ and $p_{2}$ with $k,\;\;\lambda_{1},\;\;\lambda_{2}$ and a having different values when $\alpha_{1}=\alpha_{2}=0.005$.

Comparing Figure 15, Figure 16, Figure 17, Figure 18 and Figure 19, we find that the attraction domain increases with the increase of market scale of products and the level of carbon emission reduction, while it reduces with the fairness concern level of the retailer. We can also find that the attraction domain increases with the increase of fairness concern level of manufacturer, although not so obviously.

From an economic point of view, the initial prices of the manufacturer and retailer should be in the basin of attraction in order to maintain market stability.

## 6. Chaos Control

According to the above numerical simulation, we can see that if the firms’ price adjustment speeds are beyond the stable region, the market will lose stability and even fall into chaos.

It is harmful to all the firms when the economic system is in chaos. In order to avert the risk, it is very advantageous to select suitable adjustment parameters to keep the system in a stable state.

**Figure 20:**
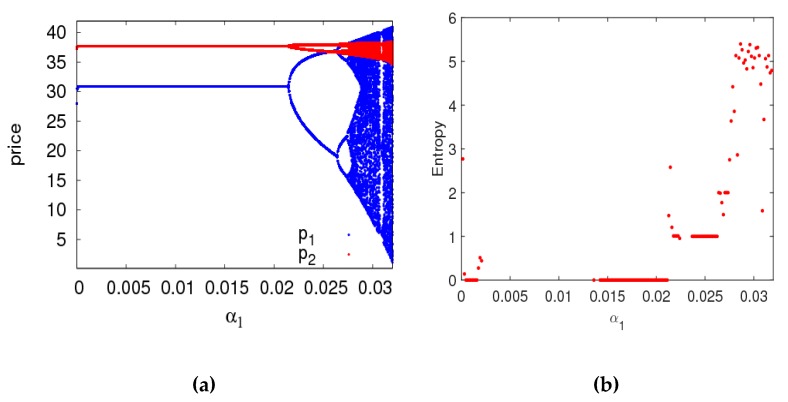
Bifurcation diagram and entropy of control system (12) with respect to $\alpha_{1}$ when v=0.5. (**a**) The bifurcation diagram, (**b**) The entropy diagram.

**Figure 21:**
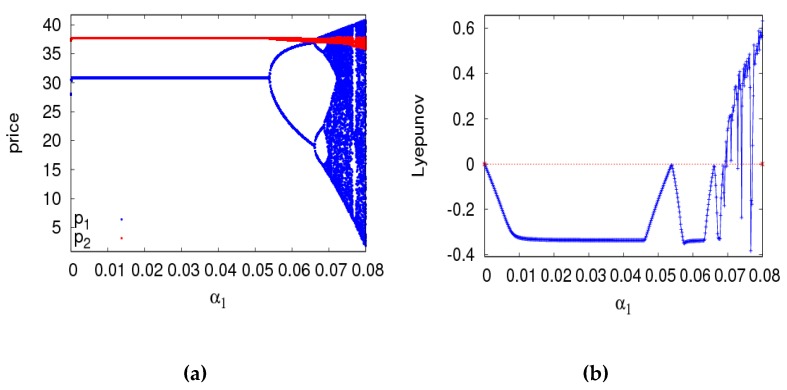
Bifurcation diagram and entropy of control system (12) with respect to $\alpha_{1}$ when v=0.8. (**a**) The bifurcation diagram, (**b**) The LLE diagram.

Scholars have proposed many methods for chaos control, such as time-delayed feedback method [46], modified straight-line stabilization method [47]. In this section, the state feedback control method is used to control the system’s chaos. The controlled system is given by:

(12) $$\{\begin{array}{c} \;p_{1}\left(t+1\right)=\left(1-v\right)p_{1}\left(t\right)+\alpha_{1}p_{1}\left(t\right)\frac{\partial U_{m}\left(t\right)}{\partial p_{1}\left(t\right)}+vp_{1}\left(t\right)\\ p_{2}\left(t+1\right)=\left(1-v\right)p_{2}\left(t\right)+\alpha_{2}p_{2}\left(t\right)\frac{\partial U_{r}\left(t\right)}{\partial p_{2}\left(t\right)}+vp_{2}\left(t\right) \end{array}$$

where v is the controlling parameter, which can be considered as the learning ability or adaptability of the manufacturer and retailer. For example, the manufacturer can adjust price decision through analyzing historical information. As what can be seen from Figure 20 and Figure 21, in the control system (12) the first bifurcation occurs at $a_{1}=0.0215$ and $a_{1}=0.052$ when v=0.5 and v=0.8 respectively. In a stable state, the system’s entropy equals zero and LLE is less than zero. In a chaotic state, the system’s entropy increases and LLE is greater than zero. Therefore, choosing an appropriate control parameter is essential for the manufacturer and retailer to delay the occurrence of price bifurcation in the dynamic system (6).

**Figure 22:**
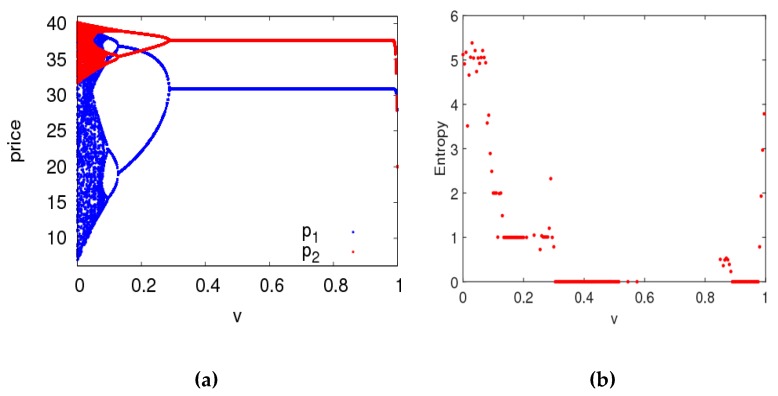
Bifurcation diagram and entropy of control system (12) with v∈(0, 1) when $\alpha_{1}=0.015,\;\;\alpha_{2}=0.008$. (**a**) The bifurcation diagram, (**b**) The entropy diagram.

Figure 22 is the bifurcation diagram and the entropy of the controlled system (12) with the change of v when $a_{1}=0.015\;\mathrm{and}\;a_{2}=0.008$. The controlled system (12) is in a chaotic state and has large entropy when 0≤v≤0.088. The controlled system (12) is in a periodic doubling bifurcation state and has low entropy when 0.088<v≤0.29. When 0.29<v≤1, the controlled system (12) returns to a stable state. Therefore, we found that the manufacturer and retailer can make the market return to a stable state with appropriate control parameter values.

## 7. Conclusions

Considering the fairness concern of the manufacturer and retailer, this paper constructs a dynamic price game model in which the manufacturer sells products through the direct channel and the traditional channel, while the retailer sells products through the traditional channel. The manufacturer considers carbon emission reduction and the retailer provides sales service. The dynamic behaviors of the dynamic price game model are analyzed using bifurcation, basin of attraction, chaotic attractors, etc. The average utilities of the manufacturer and retailer are described when parameters change. The state feedback control method is used to control the system’s chaos. The following conclusions can be obtained.

(1) The fairness concern of the manufacturer has little effect on the stability region of the system, while that of the retailer reduces the stability region of the system. The CER of the manufacturer can shrink the system’s stability region, increasing customers’ preference for the direct channel can reduce the price adjustment stable range of the direct channel and expand that of the traditional channel. The manufacturer and retailer should pay attention to the size of CER and fairness concern level in order to keep the system in a stable state.
(2) The system will enter a chaotic state through flip bifurcation with the increase of the price adjustment speed. In a chaotic state, the average utilities of the manufacturer and retailer all decline, while that of the retailer declines even more. In a stable state, the manufacturer should improve the customers’ preference for the direct channel and the retailer should choose the appropriate fairness concern level to achieve the maximum utility. Selecting appropriate control parameters, the system can return to a stable state from chaos using the state feedback control method.

However, the dynamic price game model built in this paper discards many factors that affect the dynamic decision-making of the supply chain, such as the low-carbon behaviors of customers in the closed-loop supply chain, which will affect the actual situation. Second, considering the impact of customer returns on demand will help determine whether the current results will continue. These problems will be studied in our future research.
